# Combination of Laparoscopic Salpingectomy and Endometrial Ablation: A Potentially Underused Procedure

**DOI:** 10.1089/gyn.2020.0097

**Published:** 2021-02-10

**Authors:** Florencia Greer Polite, Mary DeAgostino-Kelly, Greg J. Marchand

**Affiliations:** ^1^ Division of General Obstetrics and Gynecology Department of Obstetrics and Gynecology, Perelman School of Medicine, University of Pennsylvania, Philadelphia, Pennsylvania, USA.; ^2^ Marchand Institute for Minimally Invasive Surgery, Mesa, Arizona, USA.

## Abstract

Despite the advantages of a decreased risk of epithelial-cell ovarian cancer and the extremely minimally invasive nature of the procedure, combined salpingectomy and endometrial ablation is a potentially underused procedure in the United States to treat abnormal uterine bleeding and desired sterilization. The lack of utilization of this combined procedure might be based on factors other than clinical considerations, including slow acceptance and adoption of Committee Opinions expressing the value of salpingectomy over sterilization. Committee Opinions and randomized clinical trials have demonstrated the benefit of salpingectomy for sterilization and epithelial-cancer risk reduction, and there could be an additional protection against postablation tubal sterilization syndrome. This Commentary discusses the advantages and rationale for consideration of expanding usage of the combined approach.

## Introduction

A surgical procedure combining endometrial ablation with laparoscopic salpingectomy can have multiple benefits for patients. In select cases, the combination might prevent the need for hysterectomy, and thus avoid the risks and complications that could arise from the more-radical surgical intervention. After seeing high demand, and performing a large number of combination laparoscopic salpingectomy and endometrial ablation procedures in some of their own practices, the current authors analyze the available data on the combination procedure in an effort to determine if the procedure could present an opportunity for increased utilization in clinical practice today.

Although salpingectomy techniques are not new to benign gynecology, since the release of Committee Opinion #620^1^ in 2015—later replaced by Committee Opinion #774,^2^—there has been a steady trend toward salpingectomy,^3^ rather than tubal ligation, for patients desiring sterilization due to the added benefit of the decreased risk of epithelial-cell ovarian cancer.^4,5^ Prior to this time, it was likely that most gynecologists considered salpingectomy as the appropriate treatment for ectopic pregnancy and, otherwise, rarely performed salpingectomy outside of correcting failed tubal-ligation procedures.^6^ Although other researchers have advocated for the superiority of salpingectomy over tubal ligation for sterilization,^5,7^ the current authors felt that there was no significant literature discussing the utility of combination salpingectomy and endometrial ablation procedures.

## Endometrial Ablation

Endometrial ablation is not a perfect surgical remedy for abnormal uterine bleeding (AUB). This procedure has several drawbacks, including a small percentage of women whose bleeding pattern may actually worsen as a result of the procedure.^8,9^ In addition, researchers have noted that the procedure can have a failure rate that increases with time, making the procedure a less-attractive option for women who are further away from menopause.^10^ Nonetheless, endometrial ablation can provide many benefits to premenopausal women with heavy menstrual bleeding (HMB), including bleeding improvement rates approaching 92% and secondary amenorrhea rates approaching 58% in select populations.^11^ As a result, some clinicians may see a combination salpingectomy and ablation procedure to be an alternative to hysterectomy, with only a fraction of the recovery time.

Today, endometrial ablation is the most common procedure performed for treating AUB bleeding in the world, with ∼40,000 procedures performed each year in the United States alone.^12^ Techniques may vary from labor-intensive “resectoscopic” techniques to commercial nonresectoscopic techniques that are extremely fast and widely performed. The technique is indicated for AUB in pre- or perimenopausal females but, since the technique's inception, device manufacturers have consistently pushed that definition with direct-to-patient marketing. As a result, it can be difficult for a clinician to determine if a patient who presents desiring an endometrial-ablation procedure truly has HMB that interferes with her quality of life or wishes to cease menstruation. Nevertheless, a very high percentage of women remain satisfied with the procedure postoperatively, with a reported satisfaction rate in the range of 77%–96%.^13^ Few complications are reported, with some researchers citing a complication rate of 4.4%.^13^ The most-frequent complications reported are hemorrhage (2.4%) and uterine perforation (1.5%)^13^

## Salpingectomy

One constant requirement for endometrial ablation remains the need for long-term reliable contraception. As a result, many endometrial-ablation procedures are combined with sterilization procedures to achieve both desired outcomes simultaneously. Previously, a hysteroscopic solution was acceptable with blockage of bilateral fallopian tubes via hysteroscopy in combination with endometrial ablation in a single surgery. With the removal of Essure^TM^ from the U.S. market at the end of 2018, a purely hysteroscopic approach is no longer an option for women in the United States.^14^

Another perceived drawback to salpingectomy is that it could be seen as a less minimally invasive procedure than tubal-occlusion procedures. This is a misconception. In the hands of an experienced surgeon, laparoscopic salpingectomy may actually be a less-invasive surgery than laparoscopic tubal ligation, secondary to the extremely small size of the power instruments used to perform salpingectomy. Both bipolar and ultrasonic-energy devices are available in 5-mm or smaller sizes to complete the procedure. Extremely cosmetic techniques have been described including purely umbilical single-port techniques and 2-port techniques that hide a 5-mm incision below the pubic hairline. The procedure can be performed with reliably low blood loss, and some researchers report operative times as low as 5 minutes.^15^ This compares well to many variations of tubal-occlusion techniques, which may require larger, more-invasive entry ports. Some examples include the 8-mm devices required to place Filshie^TM^ and Hulka^TM^ clips for laparoscopic tubal occlusion.^16,17^

## Postablation Tubal Sterilization Syndrome

Adding to the appeal of salpingectomy over tubal ligation is a controversial syndrome called postablation tubal sterilization syndrome (PATSS). Many researchers have written about this syndrome of severe pain, generally starting 5–40 months after endometrial ablation, in patients who have undergone previous tubal ligations. This syndrome was first described by Townsend et al. in 1993,^18^ as a syndrome of pain that is theorized as secondary to either a buildup of blood from a small remaining portion of functional endometrium and/or an increase in uterine scarring after ablation. The incidence is not completely understood, and reports vary in limited available studies, with most reporting in the range of 6%–8%, depending on the method of tubal sterilization and the method of endometrial ablation.^19^ Some studies performed suggested a higher incidence in specific populations.^20^ The current authors were unable to find any published studies or case studies that reported an occurrence of PATSS in any patients who had undergone bilateral salpingectomy, although most salpingectomy techniques include removal of the entire fallopian tube and occlusion of the cornual segment of the fallopian tube at the time of surgery. While hysterectomy remains the gold standard of care for PATSS, many of these patients in the studies were status post endometrial ablation and laparoscopic tubal ligation.^20,21^ With studies citing salpingectomy as a possible treatment for PATTS, it stands to reason that laparoscopic bilateral salpingectomy could prevent the development of PATSS.

## Conclusions

Given the multiple advantages of the combination of laparoscopic salpingectomy and endometrial ablation, the current authors believe this combined approach is a widely underused surgery and, in some areas, could become the most-common surgery performed by gynecologic surgeons. When considering the multiple benefits, including sterility, treatment of AUB, and a decreased lifetime risk of epithelial-cell ovarian cancer, this minimally invasive technique should be included in the counseling options for appropriate patients.
